# Spotlight on early-career researchers: an interview with Natalia Castaño-Rodríguez

**DOI:** 10.1038/s42003-019-0376-8

**Published:** 2019-03-29

## Abstract

Dr. Natalia Castaño-Rodríguez is a National Health and Medical Research Council Early Career Fellow in the School of Biotechnology and Biomolecular Sciences at the University of New South Wales. Her research focuses on understanding how host immunogenetic factors interact with bacterial infection and gut dysbiosis to regulate tumorigenesis and chronic inflammation in humans, using molecular biology, microbiology, genetics, and bioinformatics. As part of our series on early-career researchers, we asked Dr. Castaño-Rodríguez to talk to us about her research and career path. She also shares the lesson she’s learned from studying the bacteria colonizing our stomachs.

**Figure:**
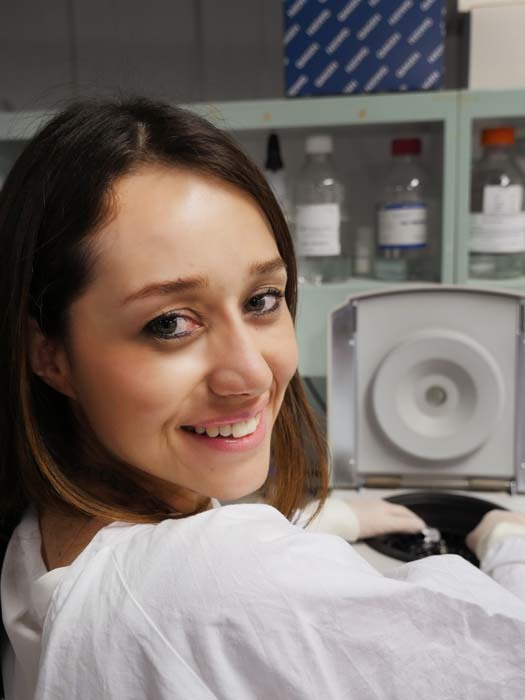
Image credit: Natalia Castaño-Rodríguez

Please tell us about your research interests

I have always been interested in immunogenetics of cancer, even as a young medical student. My first research project while I was an undergraduate student was on immunotherapy for melanoma. However, it was probably the death of a young patient presenting with pancreatic cancer who was assigned to me, that made me realize that I wanted to dedicate my life to cancer research to find ways of preventing the disease, improving the life quality, and survival rates of patients. More recently, as an early—career researcher at the University of New South Wales (UNSW Sydney), I have been investigating the interplay between host immunogenetic factors, infection with *Helicobacter* and *Campylobacter* species and gastrointestinal dysbiosis, in gastrointestinal carcinogenesis (i.e. gastric and oesophageal adenocarcinomas) and other chronic inflammatory conditions (i.e. inflammatory bowel diseases).

What has your journey been to this point?

I am currently a National Health and Medical Research Council (NHMRC) Early Career Fellow in the School of Biotechnology and Biomolecular Sciences at UNSW Sydney. However, my story started many years ago following the completion of my M.D. at Del Rosario University (Colombia), when I decided to leave my home country and move to Australia to pursue my dream of becoming a clinician researcher. In 2009, I was awarded a scholarship from COLFUTURO to undertake an M.Phil. degree in Biochemistry and Molecular Genetics at UNSW Sydney. After being awarded the University International Postgraduate Award, I initiated my doctoral studies at UNSW to investigate the role of innate immunity in *Helicobacter pylori*-related gastric cancer, which I completed in December 2014. More recently, as a research fellow, I have been working in the *Helicobacter* and *Campylobacter* Laboratory at UNSW Sydney, supported by the NHMRC and Cancer Australia. Obtaining this financial support has allowed me to do what I love, and I am deeply grateful for all these opportunities.

What are your predictions for your field in the near future?

The interactions across host genetic variability, microbial factors and environmental influences, contribute to the pathogenesis of gastrointestinal cancers and chronic inflammatory conditions. With technological advances in mind, I believe the integrated application of multi-omics, including genome, transcriptome, proteome and metabolome analyses, will be a prerequisite for identifying robust biomarkers and therapeutic targets in the near future.

Can you speak of any challenges that you have overcome?

It is widely known that when you dedicate your life to research there is a lot of uncertainty around funding and that getting support as an early career researcher is becoming increasingly difficult. This scenario can be attributed to many factors such as an increasing number of grant applications per year, less government funding towards research and, sometimes, biased review processes (i.e. there is an imbalance in funding towards more established researchers because funding bodies have a tendency to take less risk by supporting established ideas). The good news is that some important grant schemes in Australia are changing their procedures to tackle this imbalance.

In addition, when I commenced my research career, I was convinced that excelling at research was enough but now I know that I must also be successful at networking, teaching, accounting, management and even marketing. All these responsibilities can be sometimes overwhelming. However, we get better with time, more skillful, we also learn to say no, and with good mentorship, we become wiser.

From a personal point of view, I believe it can be sometimes a challenge to have a balanced life, especially for young female researchers. It is important to teach our early career researchers how to have fulfilling lives (lives that include family, friends, hobbies and other activities that lead to well-being) without compromising research output.

What advice would you give to your younger self?

I would tell myself “Natalia, you need to be truly passionate about your research to be successful, given the constant demand of this career. You also need to have the ability to accept critical assessment of your track record, ideas and work, given that you will be constantly evaluated, and at times challenged, by others. It is also important to remember that other qualities that come very handy for researchers are possessing good communication and time management skills, so take time to develop these skills as early as possible”.

What lessons did you learn from your own research, which has shaped your way of thinking?

For many years, it was believed that the stomach was a sterile environment and that *H. pylori* was the only bacterium able to colonise it, dictating its pathological outcome. However, we now know that this environment is colonized by many other bacteria that might play a significant role in health and disease. Don’t be too quick to disregard an apparent background noise, it might be in fact an interesting song!

*This interview was conducted by Associate Editor Jung-Eun Lee*.

